# Heterologous Expression of the Phytochelatin Synthase *CaPCS2* from *Chlamydomonas acidophila* and Its Effect on Different Stress Factors in *Escherichia coli*

**DOI:** 10.3390/ijerph19137692

**Published:** 2022-06-23

**Authors:** Silvia Díaz, Ángeles Aguilera, Carolina G. de Figueras, Patricia de Francisco, Sanna Olsson, Fernando Puente-Sánchez, José Eduardo González-Pastor

**Affiliations:** 1 Department of Genetics, Physiology and Microbiology, Faculty of Biology, C. José Antonio Novais, 12, Universidad Complutense de Madrid (UCM), 28040 Madrid, Spain; 2 Department of Molecular Biology, Centro de Astrobiología (CSIC-INTA), Carretera de Ajalvir, km 4, Torrejón de Ardoz, 28850 Madrid, Spain; aguileraba@cab.inta-csic.es (Á.A.); gonzalezfc@cab.inta-csic.es (C.G.d.F.); pdefrancisco@cab.inta-csic.es (P.d.F.); gonzalezpje@cab.inta-csic.es (J.E.G.-P.); 3 Department of Forest Ecology and Genetics, Forest Research Centre (INIA, CSIC), Carretera de La Coruña, km 7.5, 28040 Madrid, Spain; sanna.olsson@inia.csic.es; 4 Department of Aquatic Sciences and Assessment, Swedish University of Agricultural Sciences, Lennart Hjelms väg 9, 756 51 Uppsala, Sweden; fpusan@gmail.com

**Keywords:** phytochelatins, heavy metal resistance, stress response, oxidative stress, bioaccumulation

## Abstract

Phytochelatins (PCs) are cysteine-rich small peptides, enzymatically synthesized from reduced glutathione (GSH) by cytosolic enzyme phytochelatin synthase (PCS). The open reading frame (ORF) of the *phytochelatin synthase CaPCS2* gene from the microalgae *Chlamydomonas acidophila* was heterologously expressed in *Escherichia coli* strain DH5α, to analyze its role in protection against various abiotic agents that cause cellular stress. The transformed *E. coli* strain showed increased tolerance to exposure to different heavy metals (HMs) and arsenic (As), as well as to acidic pH and exposure to UVB, salt, or perchlorate. In addition to metal detoxification activity, new functions have also been reported for PCS and PCs. According to the results obtained in this work, the heterologous expression of *CaPCS2* in *E. coli* provides protection against oxidative stress produced by metals and exposure to different ROS-inducing agents. However, the function of this PCS is not related to HM bioaccumulation.

## 1. Introduction

Phytochelatins (PCs) are cysteine-rich small peptides, typically with the structure (γ-glutamate–cysteine)_n_–glycine, where *n* is between 2 and 11, often within 2–5. Moreover, in some plants, the C-terminal Gly can be replaced by serine, glutamine, or glutamate [1,2]. First discovered in the fission yeast *Schizosaccharomyces pombe* [3] and in cell cultures of the plant *Rauvolfia serpentine* [2], PCs have been found in all vascular plants [4,5,6] and algal groups [7,8,9,10,11], as well as in a number of fungi (including lichens), animals (for example in the nematode *Caenorhabditis elegans*), and different groups of protists [4,6,12,13,14]. In eukaryotes, cysteine thiol groups can chelate various metal(loid)s and sequester them in vacuolysosomal compartments [2,6], thus drastically reducing metal(loid) toxicity. PCs are enzymatically synthesized from reduced glutathione (GSH) by cytosolic enzyme phytochelatin synthase (PCS), a γ-glutamyl-cysteine dipeptidyl transpeptidase [15,16], belonging to the papain-like cysteine proteases [6,17,18]. The sequence alignment of PCS enzymes reveals a high degree of similarity in the N-terminal domain, whereas the C-terminal region turns out to be extremely variable. The N-terminal core domain has been reported to confer the PCS activity, containing the active catalytic site (including a conserved cysteine/histidine/aspartate triad), whereas C-terminal ensures better protein stability, regulates activity, and modulates the response to a broader spectrum of HMs [6,17,18,19,20].

PCS is constitutively expressed; however, its expression is enhanced when exposed to metal(loid) ions [16]. The catalytic site of the enzyme binds tightly to complexes formed by glutathione, in its reduced form (GSH) or its direct thiol derivatives, and metal(loid)s such as cadmium (Cd), lead (Pb), mercury (Hg), arsenic (As), copper (Cu), zinc (Zn), and iron (Fe) [2,4,6,21]. Glutathione is the most abundant reservoir of low molecular weight thiol groups in all mitochondria-bearing eukaryotes. In plants, GSH is involved in a large number of cellular processes, including defense against reactive oxygen species (ROS) [22,23], sequestration of HMs [1,24], and detoxification of xenobiotic compounds [25].

Among prokaryotes, PCS enzymes with sequence homologies to those of plants have been found in some cyanobacteria and *β*- and *γ*-*proteobacteria* [4]. So far, the small number of studies performed on PCSs from cyanobacteria suggests that this enzyme is a “half-size” protein with a high degree of homology to the N-terminal domain of eukaryotic PCSs [26]. However, unlike the PCSs of plants and other eukaryotes, in cyanobacteria, the enzyme only acts as a peptidase, thus regulating the catabolism of GSH conjugates by cleaving the glycine residue of GSH [27,28]. In fact, no differences have ever been detected in the PCS activity of cyanobacteria under metal stress compared with untreated controls [20,26,28].

**Figure 1:**
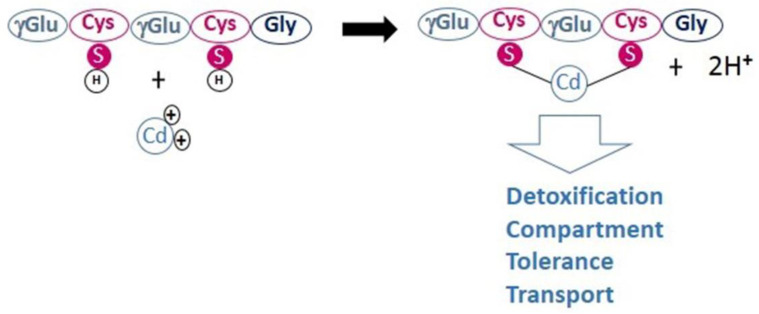
Example of phytochelatin–metal complexes for divalent cations. Figure inspired from that published by Dennis et al., 2019 [31].

PCs are able to chelate metal cations via the thiol groups present on the side chain of the cysteine residues (Figure 1). It has been suggested that HM cations bind up to four sulphur groups from one or more PCs, within an HM-PC complex [29]. Because of their high thiol (-SH) content due to Cys residues, PCs have a strong metal binding capacity that increases with the size of the PC [29,30]. In addition to glycine, terminal amino acid residues, such as glutamine, glutamic acid, serine, alanine, and β-alanine, can also occur in PCs. In total, nine different PC variants are known to date [31].

In general, the substrate for enzyme PCS is the glutathione-chelated metal ion complex. Because glutathione is usually present in high cytosolic concentrations in cells, any influx of metal ions will lead to GSH–HM complexes [32]. The increase in PCs levels in the presence of HMs may also be an indirect effect. PCs have also been shown to scavenge hydrogen peroxide and superoxide radicals, suggesting that they counteract oxidative stress [29,33,34].

The structure, biosynthetic pathways, and regulation of PCS activity have been extensively reviewed in various organisms [29,35,36,37,38], with most studies focused on plants, as well as on animals, yeasts, and bacteria. PCSs from plants are assumed to have a plastidial origin, as they share high similarities with cyanobacterial PCSs [29]. However, PCs and their biosynthetic enzymes are likely to be present in all microalgae [11,39,40]. They are clearly understudied, despite the fact that microalgae form a group of photosynthetic eukaryotic microorganisms of great interest. Therefore, many metal-binding molecules in microalgae are probably unknown, and some of them might reveal their utility for phytoremediation or other biotechnological applications [41]. The massive sequencing of microalgae genomes and transcriptomes carried out in the last decade has provided a good deal of available information on potential proteins and biosynthetic pathways involved in the detoxification of HMs and other stressors [42,43].

Indeed, in addition to a PCS similar to the ones found in plants, (*CaPCS1*), the extremophile *Chlamydomonas acidophila* RT46 harbors another PCS imported from bacteria by horizontal gene transfer, *CaPCS2* [9]. This gene was first detected in our previous study where transcripts with a putative function related to stress and HM detoxification were identified in the polyextremophile microalgae *C. acidophila*, isolated from Rio Tinto (Huelva, Spain), a natural environment characterized by an extreme acidic pH and a high content of soluble metals and metalloids [44]. One of these transcripts was annotated as a glutathione gamma-glutamyl-cysteinyl-transferase (synonymous with PCS). In a later characterization as *CaPCS2*, it was shown to consist of four exons and three introns, with an ORF of 285 amino acid residues [9]. A similar gene is also found in two other extremophiles, adapted to low pH environments and high metal(loid)s solubility: the very similar *Chlamydomonas eustigma* with 97% amino acid similarity [45], and the more divergent *DaPCS1* in *Dunaliella acidophila* [9].

In this work, we provide an updated characterization and new information on the function of this phytochelatin synthase (CaPCS2). In recent years, the number of known *PCS* genes has increased; therefore, this article also provides a general review on the phylogenetic analysis of the *CaPCS2* gene. The results obtained support the hypothesis that eukaryotic *PCS* genes do not share a common origin. Furthermore, it is confirmed that *CaPCS2* is closer to PCSs from extremophilic green algae, whereas most of the known *PCS* genes from plants and other eukaryotes form a distinct clade, which includes another known *PCS* in *C. acidophila* RT46 (*CaPCS1*). The heterologous expression of the *CaPCS2* gene resulted in elevated resistance to metal(loid)s, UVB exposure, and other stress factors in an *Escherichia coli* strain. However, the function of this CaPCS2 is not related to HM bioaccumulation.

## 2. Materials and Methods

### 2.1. Bacterial Strains and Growth Conditions

The ORF of the *CaPCS2* gene from the microalgae *C. acidophila*, strain RT46, isolated from Rio Tinto (Huelva, Spain) [46], was amplified and cloned into plasmid *pdr111*, as described in Olsson et al. (2017) [9]. *Escherichia coli* strain DH5α was chemically transformed with plasmid *pdr111* containing the cloned *CaPCS2* gene to analyze its heterologous expression. Bacteria carrying the *pdr111* plasmid without the cloned *CaPCS2* gene were used as the negative control strain (NC). The *E. coli* negative control (NC) strain and *CaPCS2*-transformed strain (CaPCS2) were routinely grown in Luria Bertani (LB) medium (Conda Laboratories) at 37 °C. For the growth curve tests, the LB medium was buffered with 100 mM MOPS [3-(N-morpholino) propane sulfonic acid] at pH 7.0. To prevent plasmid loss, in both transformed strains, the growth medium was supplemented with 100 μg mL^−1^ ampicillin (Ap).

### 2.2. Analysis of Exposure to Metal(loid)s, Salt, and Acid pH by Growth Curve Tests

The exposure to various stress agents, such as metal(loid)s, and the presence of salt and acidic pH, was analyzed by bacterial growth curves, measuring the optical density at 600 nm (OD_600_). The HM assays performed were as follows: Cu (CuSO_4_ · 5 H_2_O, Sigma-Aldrich, Madrid, Spain) at 1 and 4 mM, Co (CoSO_4_ · 7 H_2_O, Sigma-Aldrich, Madrid, Spain) at 0.5 and 1 mM, and Ni (NiCl_2_, Sigma-Aldrich, Madrid, Spain) at 0.2, 0.5 and 1 mM. In addition, the two most frequent chemical states of arsenic, trivalent arsenic (arsenite) and pentavalent arsenic (arsenate), were tested. Arsenite [As(III)] was tested as sodium (meta) arsenite (NaAsO_2_, Sigma-Aldrich, Madrid, Spain) at 0.2 and 0.5 mM, while arsenate [As(V)] was added as sodium arsenate dibasic heptahydrate (Na_2_HAsO_4_ · 7 H_2_O, Sigma-Aldrich, Madrid, Spain) at 0.5 and 1 mM.

Growth curves in the salt exposure tests were carried out in LB medium supplemented with NaCl (Sigma-Aldrich, Madrid, Spain) at a final concentration of 2%, 3.5%, or 5%. Likewise, acid pH tolerance was assessed by acidifying the LB-MOPS-Ap medium to a final pH of 6, 5, or 4.5.

All cultures were grown overnight in LB-MOPS-Ap broth, subsequently diluted and standardized to an OD_600_ of 0.01, and transferred to a sterile 96-well plate (Starstedt, Inc., Newton, MA, USA). Assays were performed at 37 °C in LB-Ap broth (untreated control) or in LB-Ap broth supplemented with different stress agents added from the beginning of the growth curve (time zero). To analyze the growth curves, OD_600_ was measured every 60 min using a microplate reader (Tecan Genios, Mannedorf, Switzerland) at 37 °C, shaking at 150 rpm, for 50 cycles (49 h). In each assay, seven different clones were tested and the mean and standard deviation were calculated.

### 2.3. Test for Resistance to UVB Radiation and Exposure to Perchlorate Using the Droplet Test

Bacterial strains were grown overnight in LB-Ap broth at 37 °C. Afterwards, the optical density of each culture was measured at 600 nm (OD_600_) and adjusted to 1.0, followed by serial dilutions. For the perchlorate assays, the minimum inhibitory concentration (MIC) was determined by pipetting 10 μL of each dilution (1–10^−5^) into an LB-Ap solid medium containing 115 or 125 mM sodium perchlorate (NaClO_4,_ Thermo Fisher Scientific, Waltham, MA, USA). The cultures were maintained for 16–24 h at 37 °C. A control without perchlorate treatment was performed to confirm that both strains had initially been adjusted to a similar cell density and that both have a similar viability. Each experiment was repeated at least three times. 

Similarly, for UVB survival tests on cultures standardized to an OD_600_ equal to 1, serial dilutions were performed as above. From each dilution (1–10^−1^- ½- ½- ½- ½), 10 μL were pipetted into solid LB-Ap medium and exposed to UVB irradiation (254 nm) at 4 mJ cm^−2^, using the irradiation chamber BS-02 UV/VIS (Opsytec Dr. Gröbel, Ettlingen, Germany). A control was performed without exposure to radiation, and then all plates were incubated overnight at 37 °C. Each experiment was repeated at least three times.

### 2.4. Determination of Cellular Metal Concentration Using Inductively Coupled Plasma Spectroscopy-Mass Spectrometry (ICP-MS) Quantitative Analysis

For the determination of the possible intracellular accumulation of metal(loid)s in *E. coli* DH5α strains (with or without the *CaPCS2* gene), the cells were grown in liquid LB-MOPS medium containing Ap at 37 °C in a shaking incubator. The growth curves were monitored by measuring the OD_600_. In all cases, the initial OD_600_ was adjusted to a value of 0.1. Metal(loid)s were added to cultures in the early stationary phase and they were incubated for a further 1 h. The treatments tested were as follows: As(V) 1 mM; Ni 0.5, 1, and 4 mM; Cu 1, 2, and 4 mM; Cd 0.3 and 1 mM. The cultures were then washed four times with MilliQ H_2_O ultrafiltration. The washed pellets were freeze-dried, pulverized, and dissolved in H_2_O-HCl-HNO_3_-H_2_O_2_ (3:1:4:0.5) using a closed microwave digestion system for subsequent analysis by inductively coupled plasma-mass spectrometry spectroscopy. The results were expressed as mg metal(loid)s g^−1^ dry weight of the cells. To ensure that the metal(loid)s concentrations were sublethal, especially for the control strain (without the *CaPCS2* gene), cell culture samples were collected before and after metal(loid) exposure, and viable cells were counted on solid LB-Ap plates. At least three independent assays were analyzed for each treatment.

### 2.5. Statistics

T-test for Equality of Means was applied to assess the significance of the differences between the control (non-treated) and treated cells. This parametric test was later validated with a non-parametric Mann–Whitney test that confirmed the results obtained in the first one. The results were expressed as means ± standard error and statistical significance was defined as a *p* value < 0.05.

### 2.6. Gene Characterization

The description of the *CaPCS2* gene published in Olsson et al. (2017) [9] was based on combined information from transcriptomic sequences and Sanger sequenced cloned regions. However, additional sequencing and comparison to the gene model prediction in *C. eustigma* [45] led us to revise the gene characterization.

### 2.7. Phylogenetic Analysis

The updated CaPCS2 amino acid sequence was used as a query in a protein BLAST search [47] against all of the protein sequences available in GenBank (the nr database downloaded in July 2021) and the three most similar sequences were downloaded. The similarity was high only in one best hit (organism|accession number|query cover|similarity for the three most similar sequences were as following: *Chlamydomonas eustigma*|GAX77972|100%|98.16%; *Chlamydomonas* sp. UWO 241|KAG1663029|77%|48.04%; *Dunaliella salina*|KAF5828933|67%|46.90%). *CaPCS1, DaPCS1*, and selected sequences from the larger phylogeny presented in Olsson et al. (2017) [9] were added to the alignment to place the gene in a larger context. Sequences were aligned with Mafft [48] using alignment option FFT-NS-I. The data matrix was analyzed by maximum likelihood (ML) after automatic model selection using ModelFinder [49] implemented in IQTree v. 1.6.12 [50] applying 1000 ultrafast bootstrap replicates [51]. Consensus topology and support values were drawn using TreeGraph2 [52].

## 3. Results

### 3.1. Tolerance to Metal(loid) Exposure

**Figure 2:**
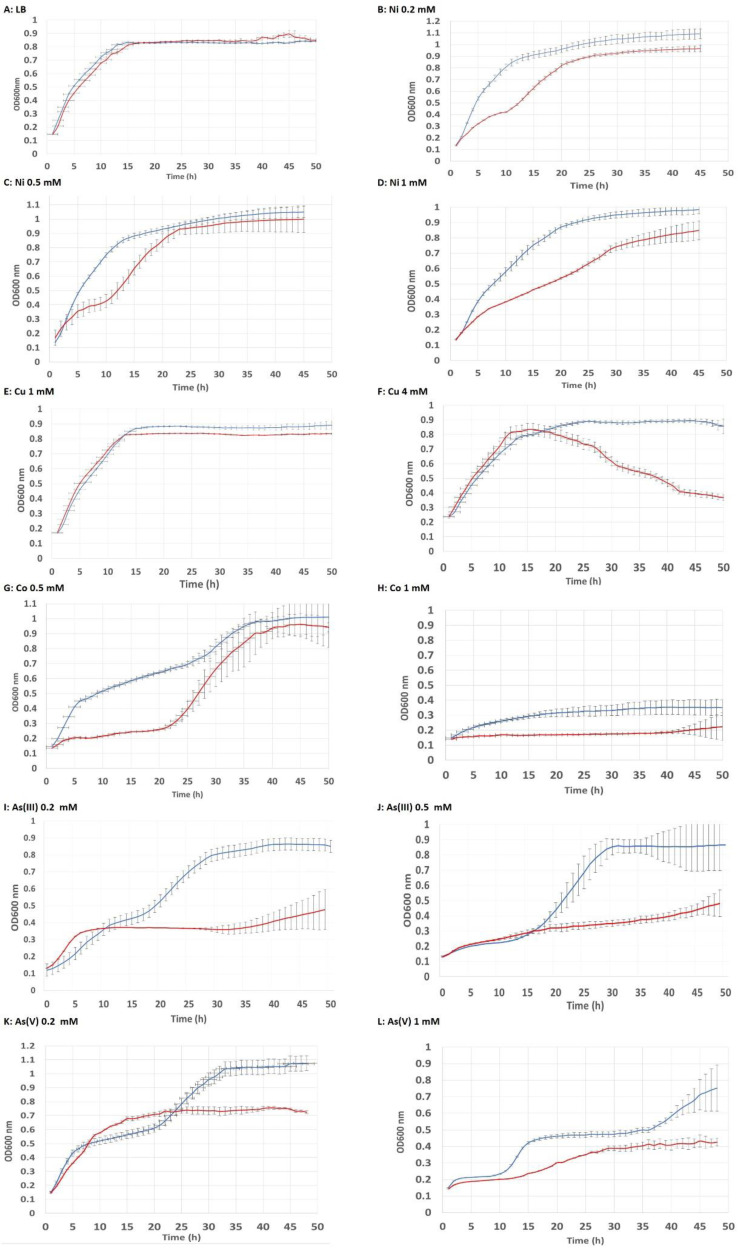
Growth curves of *Escherichia coli* in LB-MOPS broth supplemented with 100 μg mL^−1^ ampicillin (Ap). The optical density at 600 nm (OD_600_) was measured every hour for each strain for 50 cycles (49 h). NC: *E. coli* negative control (red line). CaPCS2: *E. coli* cells carrying the *pdr11* plasmid with the *CaPCS2* gene (blue line). (**A**): control not treated (LB). (**B**): NiCl_2_ 0.2 mM. (**C**): NiCl_2_ 0.5 mM. (**D**): NiCl_2_ 1 mM. (**E**): CuSO_4_ · 5 H_2_O 1 mM. (**F**): CuSO_4_ · 5 H_2_O 4 mM. (**G**): CoSO_4_ · 7 H_2_O 0.5 mM. (**H**): CoSO_4_ · 7 H_2_O 1 mM. (**I**): NaAsO_2_ 0.2 mM. J: NaAsO_2_ 0.5 mM. K: Na_2_HAsO_4_ · 7 H_2_O 0.2 mM. (**L**): Na_2_HAsO_4_ · 7 H_2_O 1 mM. The graphs show the mean of the seven different clones tested for each strain (NC and CaPCS2).

The heterologous expression of the *CaPCS2* gene in *E. coli* confers to this strain an increased tolerance to exposure to metals such as Ni, Cu, and Co, as well as to metalloids such as As, added as arsenite [As(III)] or arsenate [As(V)]. To verify that both strains, negative control (NC) strain and *CaPCS2*-transformed strain (CaPCS2), are able to reach the same maximum optical density, growth curves were previously monitored in LB-MOPS-Ap medium, without the addition of stress-producing agents, as described in the Materials and Methods section. Figure 2 shows these results expressed as mean curves and standard deviation values, obtained from at least seven different clones for both strains. In our results, we found that the heterologous expression of the *CaPCS2* gene in *E. coli* confered resistance to Ni 0.2 and 0.5 mM, and more evidently to Ni 1 mM, Co 0.5 and 1 mM, and Cu 4 mM. Furthermore, in arsenic assays, the strain expressing the *CaPCS2* gene increased its tolerance to both As(III) 0.2 and 0.5 mM, and As(V) 0.2 and 1 mM. This higher tolerance of strain CaPCS2 to metals and metalloids was observed from the beginning of the growth curve in the case of Ni and Co treatments. However, for As, both As(III) and As(V), and for Cu 4 mM, an increase in tolerance was observed from 20–22 h from the beginning of the treatment. For the Cu 1 mM treatment, no toxicity was detected in any of the strains tested, because Cu is an essential heavy metal that is not lethal at low concentrations. This assay allowed us to compare whether the highest tolerance in strain CaPCS2 was observed in the exponential phase (Ni, Co, and As) of growth or was reached in the stationary phase, in the case of Cu 4 mM.

### 3.2. Tolerance to UVB Radiation and Exposure to Perchlorate by Droplet Test

**Figure 3:**
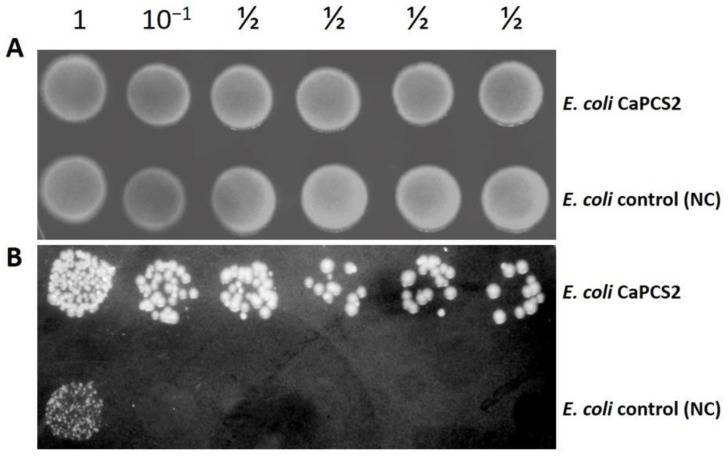
Drop test using serial dilutions of *E. coli* NC and CaPCS2 strains. (**A**): control assay with cells not exposed to UVB. (**B**): cells exposed to UVB (4 mJ cm^−2^).

Electromagnetic energy in the form of ultraviolet (UV) radiation is considered an extreme parameter [53]. The survival of the *E. coli* strain expressing the *CaPCS2* gene compared with the negative control strain was determined by exposure to UVB radiation, as described in the Materials and methods section. Figure 3 shows the result of increased survival of the CaPCS2 strain expressing the *CaPCS2* gene compared with the negative control (NC) strain after UVB exposure.

**Figure 4:**
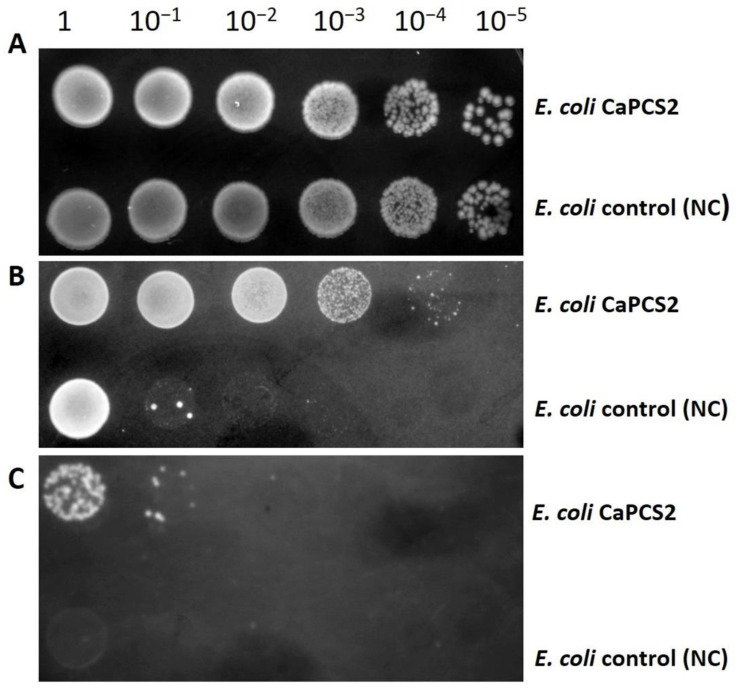
Drop test using serial dilutions of the *E. coli* negative control (NC) and CaPCS2 strains. (**A**): Control cells not exposed to sodium perchlorate (NaClO_4_). (**B**): Cells exposed to sodium perchlorate 115 mM. (**C**): Cells exposed to sodium perchlorate 125 mM.

Perchlorate is an oxidative pollutant that is toxic to many organisms by promoting the denaturation of macromolecules, oxidative stress, and DNA damage. Again, we observed a higher tolerance of the *E. coli* strain expressing the *CaPCS2* gene to exposure to all perchlorate treatments tested (115 and 125 mM sodium perchlorate) than the NC strain (Figure 4).

### 3.3. Tolerance to Acidic pH and Salt Exposure

**Figure 5:**
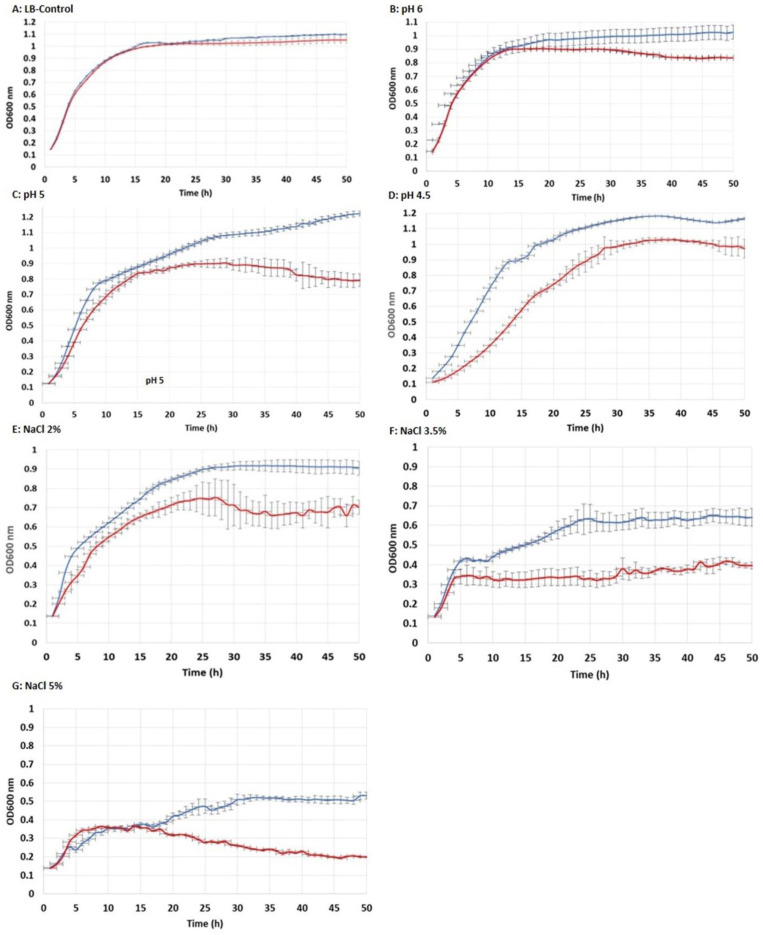
Growth curves of *Escherichia coli* in LB-MOPS broth supplemented with 100 μg mL^−1^ ampicillin (Ap). The optical density at 600 nm (OD_600_) was measured every hour for each strain for 50 cycles (49 h). NC: *E. coli* negative control (red line). CaPCS2: *E. coli* cells carrying plasmids with the *CaPCS2* gene (blue line). (**A**): Control assay (LB-MOPS-Ap) at neutral pH and NaCl 0.5%. (**B**): LB-MOPS-Ap broth at pH 6. (**C**): LB-MOPS-Ap broth at pH 5. (**D**): LB-MOPS-Ap broth at pH 4.5. (**E**): LB-MOPS-Ap broth supplemented with NaCl 2%. (**F**): LB-MOPS-Ap broth supplemented with NaCl 3.5%. (**G**): LB-MOPS-Ap broth supplemented with NaCl 5%. The graphs show the mean of the seven different clones tested for each strain (NC and CaPCS2).

Figure 5 shows the growth curves of each *E. coli* strain (CaPCS2 and NC) grown for 50 h in a buffered LB-MOPS-Ap medium at pH 7. To determine the tolerance of the strains to acidic pH, the growth curves of both strains were analyzed at acidic conditions (pH 6, 5, and 4.5), as described in the Materials and Methods section. The strain expressing the *CaPCS2* gene was able to grow to a maximum OD_600_ nm of 1.2, and tolerated acidic conditions at pH 6, pH 5, and pH 4.5. However, the control strain (NC) had a growth curve showing a lower maximum OD_600_ nm than the CaPCS2 strain, indicating a lower tolerance to acidic pH from the beginning of the growth curve.

The results of the growth curves in the salt exposure tests in the LB-MOPS-Ap medium supplemented with NaCl (Sigma-Aldrich) at a final concentration of 2%, 3.5%, or 5% showed a higher tolerance in the CaPCS2 strain than in the control (NC) strain. At the lower concentrations of 2% and 3.5%, the tolerance of the strain expressing the *CaPCS2* gene was better from the beginning of the curve. At the 5% concentration, better tolerance was observed in the CaPCS2 strain 18 h after the start of treatment.

### 3.4. Determination of Cellular Metal Concentration

To understand the possible mechanisms of metal(loid) resistance due to the heterologous expression of the *CaPCS2* gene in *E. coli*, the amount of metal or As that cells were able to accumulate was quantified by ICP-MS, as described in Section 2.4 of Materials and Methods. The concentrations tested for each metal or metalloid were sublethal, and viable cell counts were performed on LB-Ap agar plates before and after treatments. In all cases, cell density was found to be maintained at 10^8^ cells mL^−1^, which is especially important for the control strain, because it is less tolerant to the presence of these stressors. Metals or As were added at the beginning of the stationary phase of the growth curve of each *E. coli* strain to increase the possibility of accumulation inside the cell. The metal(loid) exposure time was 1 h for each of the strains (negative control and CaPCS2).

Table 1 shows the results of the bioaccumulation analysis of the tested metals and metalloids. As can be seen, the heterologous expression of the *CaPCS2* gene in the *E. coli* strain does not result in an increased cellular bioaccumulation of these metals. Statistical analysis of the data only detected significant differences in Ni 4 mM bioaccumulation, slightly higher in the NC strain than in the strain expressing the *CaPCS2* gene.

### 3.5. CaPCS2 Gene from Chlamydomonas Acidophila

**Figure 6:**
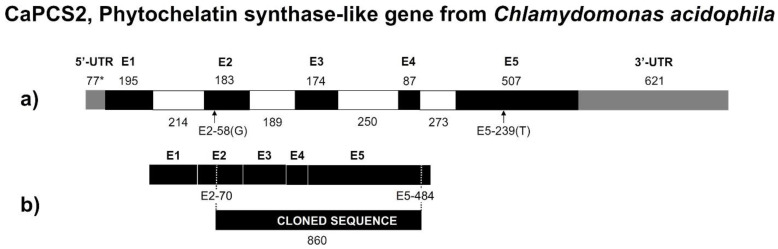
(**a**) Corrected structure of the predicted *CaPCS2* gene. Composition of exons (black, numbered E1, E2, etc.), untranslated regions (gray), and introns (white) is given in bp and drawn to scale. An asterisk indicates at least one of the base pair deletions that were erroneously present in the sequence previously described in Olsson et al., 2017 [9], the missing base is indicated in parentheses. (**b**) Coding region of the predicted *CaPCS2* (**top**) and the region cloned in *E. coli* (**bottom**).

The acquisition of additional genomic sequences and comparison with the *C. eustigma* gene prediction, for which the entire genome has been sequenced [45], allowed us to revise the gene characterization. We concluded that in the previous description, two deletions in the transcriptomic sequences erroneously caused a frame shift and a truncated ORF in the corresponding regions. The updated prediction of the *CaPCS2* gene and the cloned region in *E. coli*, after the removal of all non-coding regions (i.e., introns), is shown in Figure 6. The corresponding sequence data were submitted to GenBank (accession number ON292503).

As already observed in Olsson et al. (2017) [9], *CaPCS2* shows some intermediate characteristics to eukaryotic and prokaryotic *PCS* genes: the amino acid sequence suggests a closer relationships with certain prokaryotic than plant genes, but it is longer and has introns, like eukaryotic *PCS*. With 380 coded amino acids, CaPCS2 is longer than previously described. It is one amino acid shorter than the highly similar *C. eustigma* PCS and there are only three additional amino acid changes between them. The strictly conserved Cys residues and catalytic triad (Cys56, His162, and Asp180) described in *Arabidopsis* [18,20] are also present in CaPCS2 (Cys70, His189, and Asp207). Similar to other PCS found in bacteria [26], CaPCS2 lacks four out of the five conserved Cys residues in the N-terminal. The PCS-like enzyme from the primitive red alga *Cyanidioschyzon merolae* [54], which contains both C- and N-terminals, has an additional module in front of the N-terminal. This is, however, not a feature shared by *CaPCS2* and the most similar genes. 

**Figure 7:**
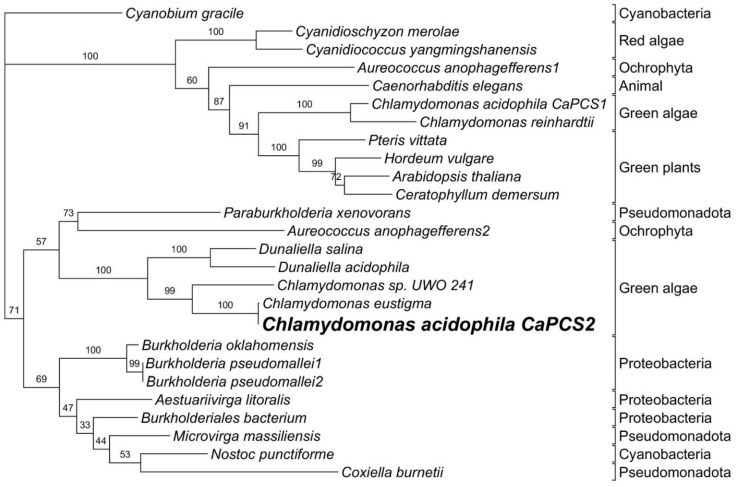
Phylogenetic relationships of *CaPCS2* and the most similar genes obtained with a protein BLAST, together with a selection of other known *PCS* genes. The tree represents the bootstrapped majority consensus IQ tree.

The combination of multiple differences and the results from the phylogenetic analysis (Figure 7) support the previous hypothesis of the eukaryotic *PCS* genes not sharing one common origin. The additional sequence information that have been made available after the initial characterization of *CaPCS2* do not change the phylogenetic position of it, but instead confirms the position of *CaPCS2* together with a few genes from other extremophilic green algae, while the majority of known *PCS* genes from plants and other eukaryota form a distinct clade, in which *CaPCS1* is included.

## 4. Discussion

### 4.1. Role of Phytochelatin Sythases in Tolerance to Abiotic Stressors

PCSs are key enzymes for the detoxification and accumulation of HMs in many organisms. The PCS enzyme is able to synthesize PCs in the presence of metal ions using GSH or related thiol peptides as a substrate [54]. The role of PCs in HM chelation has been widely described in the literature. Some authors suggest that this affinity and binding capacity for metals and metalloids is higher than in metallothioneins (MTs) [55,56,57,58]. MTs are small, cysteine-rich, metal-binding proteins that have various biological functions, which include essential metal homeostasis, HM detoxification, and cellular antioxidative defense [59,60].

The production of PCs is induced by a wide range of metals and metalloids, including As [1,61,62,63,64,65,66]. Many results show the role of PCs in the accumulation and detoxification of metals by chelating these toxic ions to form complexes. However, there is less evidence on the role of PCs in other abiotic stress responses. It is well known that exposure to HMs and arsenic leads to an increased production of ROS in cells. Similarly, exposure to various cellular stress factors such as UVB, H_2_O_2_, heat, or salinity is also directly or indirectly related to the generation of ROS. These ROS are potentially harmful to the cell, as they can increase the level of oxidative damage and thus the loss of cell structure and function. An important aspect of survival under stress conditions is the development of multiple stress tolerance mechanisms.

Genes encoding PCS have been cloned from different organisms including plants such as *Arabidopsis thaliana* (AtPCS1, AtPCS2), *Triticum aestivum* (TaPCS1), *Ceratophyllum demersum* (CdPCS1), *Lotus japonicus* (LjPCS1 and LjPCS3), *Oryza sativa* (OsPCS1), *Pteris vittata* (PvPCS1), *Brassica juncea* (BjPCS1); yeasts such as *Schizosaccharomyces pombe* (SpPCS); the nematode *Caenorhabditis elegans* (CePCS1); and microalgae such as *Chlamydomonas acidophila* (CaPCS2) [9,19,57,64,67,68,69,70,71,72,73,74]. Some of these genes encoding PCS have been functionally expressed in *E. coli*, yeasts, and plants to enhance metal accumulation and tolerance. The cloning and functional characterization of PCS genes from different species and their heterologous expression in model species provide new information about how PCSs control HM detoxification at a molecular level [62,65,75,76,77]. However, the transgenic organisms developed have had varying degrees of success and contradictory results [61,62,65,76,78,79,80,81,82,83,84,85,86,87,88]. Moreover, it has been hypothesized that these disparities in metal response in transgenic plants may be due to different modulated PCS gene activities. For example, heterologous expression of the wheat PCS gene (TaPCS1) in rice enhances Cd sensitivity [85]. Tobacco expressing NtPCS1 from Nelumbo nucifera exhibited an increased tolerance to As and Cd [89]. In higher plants, it is common to possess two PCS genes; however, only in A. thaliana has the function of both PCS genes (AtPCS1, AtPCS2) been analyzed by overexpression and heterologous expression in different organisms. According to some authors, AtPCS1 only confers metal tolerance, while AtPCS2 suggests a physiological role, apart from metal detoxification [90]. AtPCS2 shares a high homology (84%) at the amino acid level with AtPCS1, but AtPCS2 expression levels are far lower compared with AtPCS1 [19,91]. Therefore, it has been postulated that AtPCS1 is the predominant participant in PCS activity. Despite an increasing understanding of the roles of AtPCS1 in HM detoxification or tolerance, relatively few reports have documented the roles of AtPCS2. For example, in Arabidopsis, overexpression of AtPCS1 showed hypersensitivity to Cd stress [62], but enhanced As tolerance [63]. The heterologous expression of AtPCS1 in *Brassica juncea* enhanced its tolerance to As and Cd stress [70]. Furthermore, AtPCS2 was able to partially rescue the Cd hypersensitivity of the AtPCS1-deficient cad1-3 mutant. The heterologous expression of AtPCS2 in *S. pombe* and *S. cerevisiae* were shown to confer Cd tolerance [91].

On the other hand, the identification of *PC-deficient* mutants has further broadened our understanding of the roles of PCS in HM accumulation and tolerance. The *OsPCS1* mutants of *Oryza sativa* exhibit increased sensitivity to As and Cd [92].

The role of PCs may not be restricted to the chelation of potentially deleterious ions. In addition to a metal detoxification activity, new functions have been also reported for both PCS and PCs [93]. Earlier studies have shown that PCS responds to other abiotic stresses as well as HMs. In 1998, Mallik and Rai published the function of a “probable phytochelatin” in the cyanobacterium *Anabaena doliolum*, which offers tolerance to different HMs, but also provides protection against heat shock, cold shock, anaerobiosis, and UVB radiation [94]. Since then, to gain new insights into the possible roles of PCS in abiotic stress responses, the transcriptional levels of various *PCS* genes and PCs content were investigated in response to various cellular stress-producing factors such as abscisic acid (ABA), salt, drought, and cold and heat shock. The results showed that these stress-producing factors significantly increased *PCS* gene transcription or PC content in garlic, potato, or *A. thaliana* [95,96,97]. For example, the heterologous expression of the *PCS* gene of *Anabaena* in *E. coli* alleviated the damaging effects of high temperature, salinity, carbofuran, cadmium, copper, and UVB [78,98,99]. These authors showed that PCs may provide some protection against UVB radiation, suggesting an antioxidant or protein thiol-protective role. In other studies, the insertion of an extra copy of the *PCS* gene in *Anabaena* showed an approximately 22.3% increase in growth rate under UVB, NaCl, heat, CuCl_2_, carbofuran, and CdCl_2_, and it also registered five-fold higher PCs production [99]. These previous reports strongly suggest that PCS plays an important role in adaptation to abiotic stress. Moreover, both the widespread occurrence and constitutive expression of PCS throughout all organs further indicate the essential role of PCS in stress responses. 

In a previous work, cloning and heterologous expression of the *CaPCS2* gene isolated from the extremophilic microalgae *C. acidophila* in *E. coli* resulted in enhanced Cd resistance in the bacteria [9]. In the present study, it was observed that the same *E. coli* strain overexpressing the *CaPCS2* gene, also shows an increased tolerance to other metal(loid)s such as Ni, Cu, Co, As(III), and As(V) and cell stress inducing factors, such as acidic pH, increased salinity, perchlorate, and exposure to UVB radiation. All of these stress factors have in common that they all lead to an increase in ROS both directly and indirectly. It is well known that metals and metalloids can cause ROS directly by participating in the Haber–Weiss and Fenton reaction (Fe, Cu, Cr, and Co) or indirectly (Cd, Zn, Ni, and Al), though interaction with the antioxidant defense system, by alteration of the electron transport chain or through the induction of lipid peroxidation. Similar observations were made in the case of the *Anabaena PCS* gene when it was expressed in *E. coli*, providing tolerance against Cd, heat stress, and salt stress [78]. Earlier studies showed that the expression of *AtPCS1* in *E. coli* and *Saccharomyces cerevisiae* led to enhanced Cd tolerance and accumulation [57,100]. The tolerance provided by the different PCSs to this diversity of cellular stressors is, possibly, due to the ability of the cysteine sulfhydryl groups from PCs to react by scavenging free radicals, neutralizing the toxic effect of ROS [78,101,102]. These studies indicate that the *PCS* genes of photosynthetic organisms can be used to develop genetically modified organisms that are more tolerant to all of the abiotic factors that generate oxidative stress in cells. However, a complete understanding of the mechanisms of tolerance to multiple environmental stresses has not yet been achieved.

In this study, the *E. coli* strain expressing the *C. acidophila PCS* gene also showed tolerance to perchlorate. This compound is a potent oxidizing agent that induces bactericidal effects and can be found naturally in extreme arid environments such as the Atacama Desert [103]. Perchlorate is widespread in the global environment [104]. It is a ubiquitous contaminant in water, soil, and food and is toxic to most known microorganisms and humans [105]. It is a contaminant resulting from anthropogenic activity, and is used in aerospace products, as well as industrial and military applications, such as fertilizers, explosives, fireworks, and rocket fuels [106,107]. Some authors have observed that resistance to UVB is related to resistance to perchlorate exposure, perhaps due to the fact that in both cases there is a high increase in ROS produced by both abiotic stress factors [104,108]. A possible role of PCs in protection against oxidative stress may explain the role of CaPCS2 in tolerance to other abiotic stresses different to metal(loid)s. However, reports on the functions of PCs other than metal chelation are scarce, making it very difficult to draw conclusions. However, almost all reports seem to agree that *PCS* gene expression and enzymatic activity can be affected by stress in general, including acidic pH and salt exposure, as we observed in this study. Similarly, Kim et al., 2019, described that in *A. thaliana*, transcript levels of the *AtPCS2* gene were significantly increased by salt stress and, the overexpression of AtPCS2 resulted in enhanced seed germination and seedling growth of *A. thaliana* under salt stress. According to the authors, AtPCS2 in *A. thaliana* is involved in salt resistance, but it does not play a key role in HM response or tolerance [109]. The next important question is how some PCSs contribute to salt tolerance. In plants, proline is a major osmolyte, and its accumulation represents an important mechanism of a high salinity response [110]. *AtPCS2*-overexpressing plants accumulated more proline than wild type (WT) plants under salt stress. Salt exposure increases the accumulation of Na^+^ and interferes with intracellular K^+^ uptake, because the two cations compete for the same binding sites [99,111,112]. Transgenic plants overexpressing *AtPCS2*-accumulated less Na^+^ and more K^+^ compared with the WT plants. Taken together, these results suggest that AtPCS2 preferentially protects cell vitality against damage caused by high salinity by mediating osmotic adjustments and ionic homeostasis through proline accumulation and a reduced Na^+^/K^+^ ratio [109]. Regarding induction levels, *AtPCS2* gene expression was increased up to four and six-fold through treatment with 100 and 200 mM NaCl, respectively. In contrast, *AtPCS1* expression did not change in response to salt stress. These different expression patterns suggest that AtPCS2 might play a more important role in salt stress adaptation than AtPCS1 [109]. In *AtPCS2*-overexpressing plants, combined salt and H_2_O_2_ treatments resulted in reduced levels of oxidative stress markers [113,114]. Therefore, these results seem to indicate that plants overexpressing *AtPCS2* have a superior ROS scavenging capacity.

### 4.2. Phytochelatin Sythetase and Heavy Metal(loid)s Bioaccumulation

The enhanced tolerance to HMs and As in *PCS* transgenic organisms suggested that the expression of additional *PCS* might also lead to the increased accumulation of these metal(loid)s. Therefore, expressing *PCS* genes in homologous or heterologous systems may increase the production of PCs, which may lead to increased tolerance and accumulation of HMs. The overexpression of *AtPCS1*, *TaPCS1*, and *SpPCS1* has been achieved in different plants, which resulted in an enhanced production of PC peptides in transgenic lines. Again, the results from these studies were contradictory in terms of metal accumulation [63,65,70]. In plants, overexpression of the *AtPCS1* gene in *A. thaliana* [62] did not result in higher tolerance to Cd than WT plants, but overexpression of the same gene in *Brassica juncea* resulted in a high tolerance to Cd and Zn exposure, but in terms of the accumulation of these metals, the results were significantly lower than in the WT plants [70]. *Nicotiana tabacum* expressing *AtPCS1* displayed enhanced cadmium tolerance and accumulation [65] and *Nicotiana glauca* expressing *TaPCS1* (wheat PCS) has shown increased Cd and Pb tolerance and accumulation [54]. The heterologous expression of *Ceratophyllum demersum PCS (CdPCS1)* in matured *A. thaliana* plants led to a significant increase in metal(loid)s accumulation in aerial tissues relative to WT. A similar kind of mechanisms that might be involved in *CdPCS1*-expressing *A. thaliana* plants led to a higher metal(loid)s accumulation in aerial tissues [76]. Furthermore, analysis suggested that *CdPCS1* expression in rice enhanced the accumulation of As in roots, leading to a significantly low accumulation in the aerial parts including in rice grains [73]. Overall, it was estimated that 33.3% of experiments with transgenic plants overexpressing *PCS* showed a positive relationship between tolerance and Cd accumulation, while 25% evidenced a negative relationship [89]. At present, the reasons behind these contrasting effects are still unclear [115].

*AtPCS1*-deficient *A. thaliana* plants are highly sensitive to Cd, while the overexpression of *AtPCS1* changed the Cd tolerance and the ability of plants to accumulate Cd [19,65]. Tobacco plants expressing *NtPCS1* showed an increased tolerance to As and Cd, but changes in the accumulation of As and Cd were not observed [89].

The heterologous expression of the *CdPCS1* gene from *Ceratophyllum demersum* in *Arabidopsis* and *E. coli* enhanced the accumulation of HMs [76]. The heterologous expression of *CdPCS1* in *E. coli* enhanced the cellular Cd content by 23% [116]. The heterologous expression of the *A. thaliana AtPCS1* gene in *E. coli* resulted in a 50-fold increase in the accumulation of As in a non-toxic form inside the cell [81]. In contrast with the observations of these authors, our results showed that the *E. coli* strain heterologous expressing *CaPCS2* did not show a significant intracellular accumulation of the tested metals (Cu, Ni, and Co) and metalloids (As) compared with the control strain. Furthermore, in the case of Ni 4 mM treatment, a slightly higher accumulation was detected in the case of the NC strain. These results seem to confirm that CaPCS2 does not play an important role in the intracellular bioaccumulation of metals. In our previous studies, a strong induction of the *CaPCS2* gene, analyzed by RT-PCRq, was observed in *C. acidophila* exposed to Cd, As(III), or As(V). The highest gene induction of 2959-fold was observed in treatments with 5 mM As(V) for 3 h, followed by 1275-fold with 1 μM Cd 3 h and 526-fold with 1 mM As(III) [9,117]. This strong induction of *CaPCS2* by meta(loid)s may suggest that CaPCS2 might be involved in metal(loid)s resistance in *C. acidophila*. As described above, not all PCS are involved in HM bioaccumulation. In *C. acidophila*, the presence of at least two different *PCS* genes, *CaPCS1* and *CaPCS2* [9], could indicate different functions of both PCS. On the other hand, several authors have shown that in microalgae, bioaccumulation and resistance to HMs is due to the presence of both PCs and phosphorus, in particular due to the role of polyphosphate in the binding and accumulation of HMs [117,118,119,120].

An important aspect, intensively debated in the literature, concerns the structural and functional diversity of PCSs. The amino acid sequences of most eukaryotic PCSs consist of conserved N-terminal and variable C-terminal domains. The N-terminal domains from most organisms share high levels of sequence homology, and are suggested to have a catalytic activity [121]. There are several conserved Cys residues in the N-terminal domains, which may be related to the catalytic activity of PCS enzymes. The C-terminal domain, containing pairs of Cys and Glu residues, is involved in the response to metal ions. In the absence of HMs, the N-terminal domains have no enzymatic activity. When HMs are detected, the C-terminal domain forms a special structure with the HM ions that can initiate catalytic activity in the N-terminal domain. Analysis of AtPCS1 showed that the conserved N-terminal domain is necessary and sufficient for the catalytic activity of the enzyme, while the evolutionarily divergent C-terminal domain is involved in the responsiveness to a wide range of HMs [122]. The different responsiveness to a set of HMs of LjPCS1 and LjPCS3, two different PCS enzymes in *Lotus japonicus*, indicated that the different HM activation patterns between these two proteins were mainly due to differences in their C-terminal domains [72]. Differences in the C-terminal between higher plants and CaPCS2 might explain the wider range of metals that induce the latter.

To date, *PCS*-encoding genes have been isolated and functionally characterized in plants [75,123]. However, studies of these genes in other organisms, including microalgae, are more scarce, despite the fact that bioaccumulation is one of the main resistance mechanisms against metal(loid)s used by microalgae, although other protection mechanisms also exist [124]. Many studies investigated the production of microalgal PCs, induced by Cd, Hg, Zn, or As [125,126,127,128,129,130,131,132,133,134,135,136,137,138,139]. However, it is necessary to increase knowledge in this group of photosynthetic eukaryotic microorganisms, with studies such as the one carried out in this work, where the heterologous expression of a microalgal *PCS* is analyzed in a model organism such as *E. coli*, to determine its function in the face of stress produced by various abiotic agents.

Further knowledge on the function of different PCS in organisms other than plants, such as microalgae, is needed in the future. The presence of two PCS in plants and in the *C. acidophila* strain RT46, isolated from Rio Tinto (Huelva, Spain), suggests a diversity in the possible role of these enzymes in tolerance to various cellular stressors.

## 5. Conclusions

1. Heterologous expression of the *PCS* gene from *C. acidophila* (*CaPCS2*) confers resistance in *E. coli* to several metal(loid)s that directly or indirectly produce ROS. This PCS is not involved in the bioaccumulation of metal(loid)s in the cell.
2. The *E. coli* strain transformed with the *CaPCS2* gene showed increased resistance to exposure to various cellular stress factors, such as the presence of perchlorate and exposure to UVB radiation. This strain also exhibited greater tolerance to the presence of salt and acidic pH in the culture medium than the control strain.
3. The phylogenetic analysis of the *CaPCS2* gene showed characteristics intermediate to eukaryotic and prokaryotic *PCS* genes. The amino acid sequence suggests closer relationships to bacterial PCS than to plant PCS. However, the complete gene sequence contains introns, such as eukaryotic genes.
4. The strictly conserved Cys residues and catalytic triad (Cys56, His162, and Asp180) are also present in CaPCS2 (Cys70, His189, and Asp207). CaPCS2 lacks four of the five conserved Cys residues at the N-terminus, such as bacterial PCS.

The position of *CaPCS2* is most closely related to extremophilic green algae, whereas most known *PCS* genes from plants and other eukaryotes form a distinct clade. The results of the phylogenetic analysis support the hypothesis that eukaryotic *PCS* genes do not share a common origin.

## Figures and Tables

**Table 1:** Bioaccumulation of metal(loid)s in cells of *E. coli* NC and CaCPS2 strains quantified by ICP-mass technique expressed in mg g^−1^ dry weight (d.w). Data expressed as the arithmetic mean of at least three assays. SD: standard deviation. Exposure time: 1 h.

Metal/	Concentration (mM)	Bioaccumulation		Bioaccumulation		*t*-Test
Metalloid		(mgg^−1^d.w)		(mgg^−1^d.w)		For Equality ofMeans
		NC	SD	CaPCS2	SD	Sig(2-tailed)
	1	6299	1814	6624	2734	0.902
Cu	2	1006	116	1111	59	0.232
	4	3822	1192	4204	1536	0.504
Cd	0.3	4653	893	4640	1294	0.982
	1	13,377	3461	14,547	2773	0.533
As	1	246	63	272	70	0.513
	0.5	381	25	338	148	0.642
Ni	1	1375	102	1017	200	0.051
	4	8486	1099	6234	1365	0.011 *

Cellular bioaccumulation tests for metals and metalloids. Comparison between the accumulation of the negative control strain NC and the strain expressing the *CaPCS2* gene. The asterisk (*) indicates significant differences between NC and CaPCS2 data.

## Data Availability

The data are available from the corresponding author upon reasonable request.
